# Characterization of Fatty Acid Metabolism-Related Genes Landscape for Predicting Prognosis and Aiding Immunotherapy in Glioma Patients

**DOI:** 10.3389/fimmu.2022.902143

**Published:** 2022-07-12

**Authors:** Feng Jiang, Fei Luo, Ni Zeng, Yan Mao, Xinfang Tang, Jimei Wang, Yifang Hu, Chuyan Wu

**Affiliations:** ^1^ Department of Neonatology, Obstetrics and Gynecology Hospital of Fudan University, Shanghai, China; ^2^ Department of Dermatology, Affiliated Hospital of Zunyi Medical University, Zunyi, China; ^3^ Department of Pediatrics, The First Affiliated Hospital of Nanjing Medical University, Nanjing, China; ^4^ Department of Nephrology, The Affiliated Lianyungang Oriental Hospital of Xuzhou Medical University, The Affiliated Lianyungang Oriental Hospital of Kangda College of Nanjing Medical University, The Affiliated Lianyungang Oriental Hospital of Bengbu Medical College, Lianyungang, China; ^5^ Department of Geriatric Endocrinology, The First Affiliated Hospital of Nanjing Medical University, Nanjing, China; ^6^ Department of Rehabilitation Medicine, The First Affiliated Hospital of Nanjing Medical University, Nanjing, China

**Keywords:** fatty acid metabolism, tumor microenvironment, prognosis, signature, glioma

## Abstract

Glioma is a highly malignant brain tumor with a poor survival rate. The involvement of fatty acid metabolism in glioma was examined to find viable treatment options. The information was gathered from the Cancer Genome Atlas (TCGA) and the Chinese Glioma Genome Atlas (CGGA) databases. A prognostic signature containing fatty acid metabolism-dependent genes (FAMDs) was developed to predict glioma outcome by multivariate and most minor absolute shrinkage and selection operator (LASSO) regression analyses. In the TCGA cohort, individuals with a good score had a worse prognosis than those with a poor score, validated in the CGGA cohort. According to further research by “pRRophetic” R package, higher-risk individuals were more susceptible to crizotinib. According to a complete study of the connection between the predictive risk rating model and tumor microenvironment (TME) features, high-risk individuals were eligible for activating the immune cell-associated receptor pathway. We also discovered that anti-PD-1/PD-L1 and anti-CTLA4 immunotherapy are more effective in high-risk individuals. Furthermore, we demonstrated that CCNA2 promotes glioma proliferation, migration, and invasion and regulates macrophage polarization. Therefore, examining the fatty acid metabolism pathway aids our understanding of TME invasion properties, allowing us to develop more effective immunotherapies for glioma.

**Graphical Abstract:**
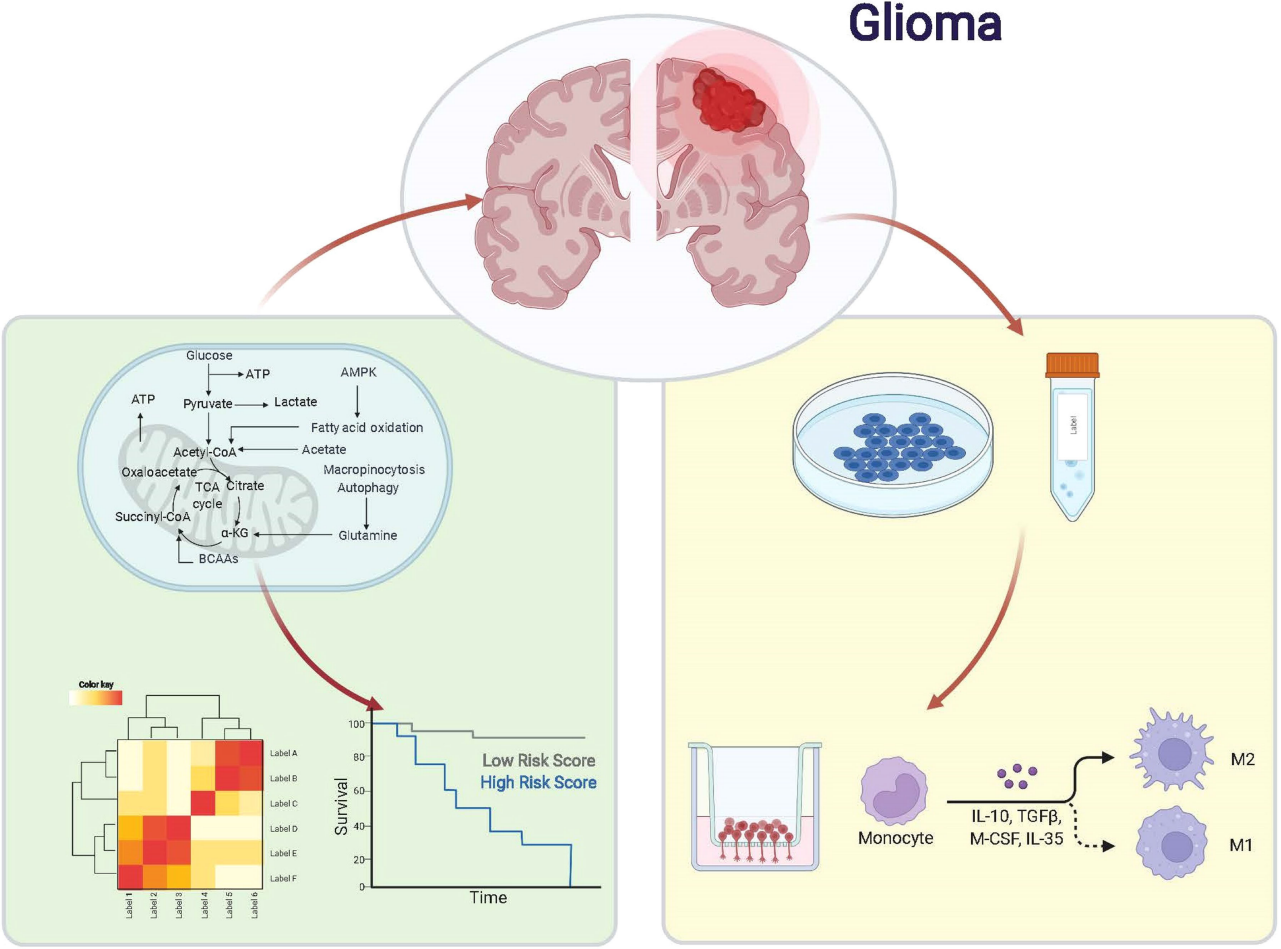


## Introduction

Glioma is the most prevalent type of primary brain tumor and accounts for almost 80% of the central nervous system (CNS) malignancies (1). Although there are mounting options for treating glioma, mainly comprising surgery, chemotherapy, and immunotherapy, the prognosis is still not ideal, with a median survival of 8 months for glioma patients by CBTRUS data (2). At present, glioma patients’ treatment options mainly depend on WHO classification (WHO I-IV) (3, 4), as well as molecular subtypes, like IDH mutations, 1p19q deletion status, and MGMT methylation (5–7). Nevertheless, there are still significant differences in drug resistance, recurrence rates, and survival times for glioma patients with the same subtype. Available biomarkers cannot provide glioma patients a personalized treatment and optimal survival time. Therefore, identifying novel targets for the glioma therapy approach and prognostic assessment is warranted.

Different from normal cells, cancer cells have distinct metabolic features. When the carcinogenic signal is inhibited, they cope with unfavorable microenvironments by changing their metabolism to preserve cancer cell proliferation and survival (8–10). With a deep understanding of tumor biology and the complexity of tumor metabolism, modern metabolic reprogramming is a sign of a malignant tumor. Cancer cells and tumors have a lot of metabolic variabilities compared to normal tissues, but essentially little metabolic activity is unique to tumors. And there is metabolic heterogeneity between different tumors (11, 12), which leads to the difference in the efficacy of metabolic drugs in other tumors. Metabolic characterization and metabolic reliance change as cancer progress from premalignant lesions to regional invasion and metastasis (13, 14). In recent years, the link between fatty acid metabolism and tumor development has been a popular topic. Fatty acid metabolism is critical for tumor cell proliferation and spread. In terms of synthesis, fatty acids can participate in the structural synthesis of phospholipids on the cancer cell membrane and the transduction of necessary signals (such as PI3K/Akt/mTOR) (15, 16). In terms of decomposition, cancer cells mainly use fatty acids β- ATP produced by oxidation to maintain the required energy, and nicotinamide adenine dinucleotide phosphate (NADPH) is used to maintain the redox balance in the body (17). Increased lipolysis and fatty acid production induced by the activated nuclear factors-B (NF-B) signal has been demonstrated to cause lymph node metastases in cervical cancer patients (18). Through remodeling, active fatty acid oxidation may help AML cells survive and bone marrow adipocytes lipolyze (19). Furthermore, the expression pattern of genes involved in fatty acid metabolism is linked to the degree of malignancy, prognosis, and immunophenotype of glioma (20). The function of fatty acid metabolic features in the therapeutic therapy of gliomas, on the other hand, has not been well investigated.

**Figure 1:**
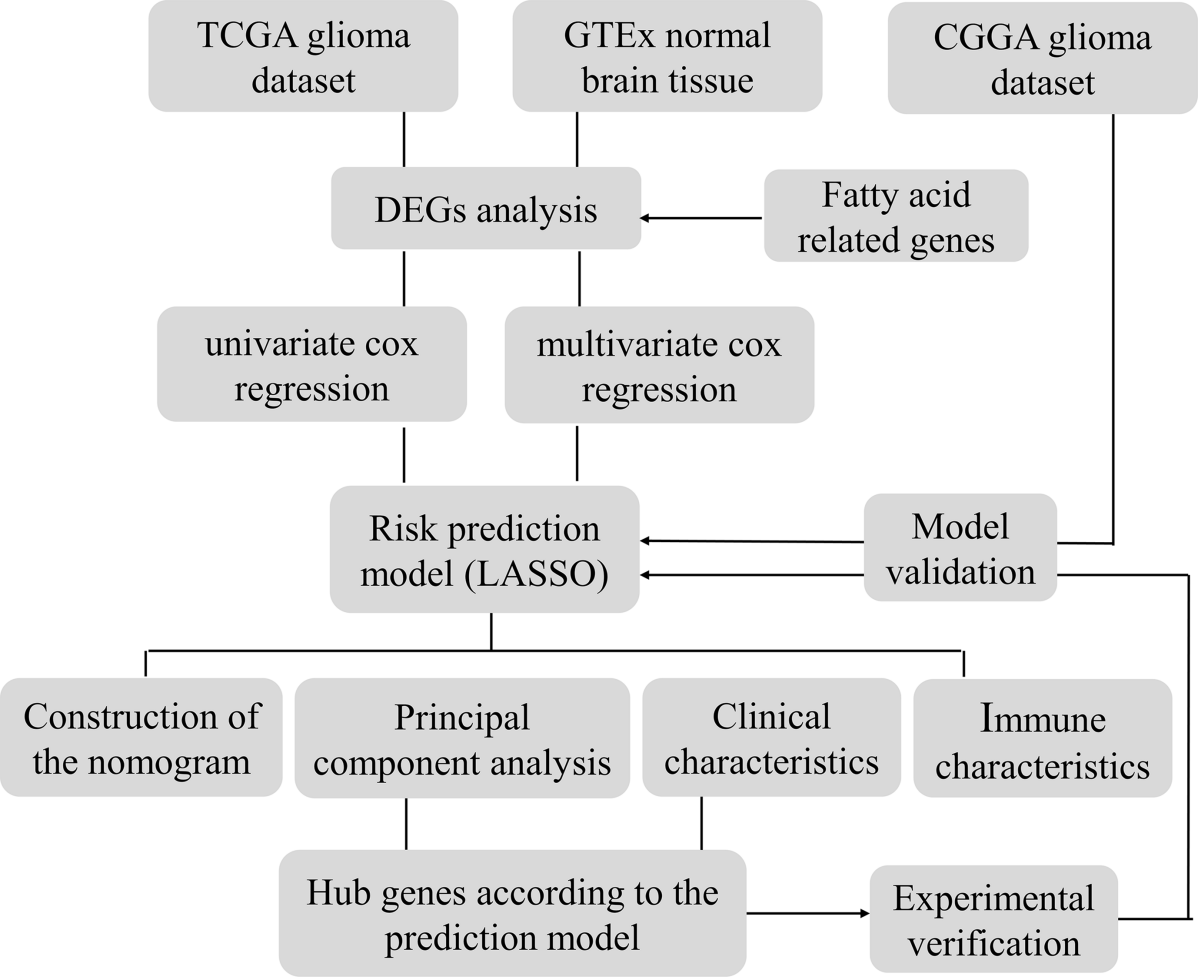
Flowchart.

The genetic information of glioma samples was analyzed using two databases to thoroughly estimate the fatty acid metabolism model and create the dependent predictive risk score model. And the survival result of glioma patients was independently predicted using the constructed scoring model. The relationship between the TME cell infiltration characteristics and the prediction risk rating model was also looked into. The predictive risk score approach correctly identifies glioma patients who are immunotherapy candidates, indicating that maybe fatty acid metabolism would be an essential individual TME feature for glioma development. These findings bring up new research pathways into the metabolic process of glioma and its treatment (Graphic Abstract, **Figure 1** Flowchart).

## Materials and Methods

### Downloading and Analyzing Data

TCGA database (https://portal.gdc.cancer.gov/) was applied to retrieve raw RNA sequencing (RNA-seq) data profiles for 701 glioma tissue samples. The TCGA database also yielded clinical information on 668 glioma samples, including age, gender, grade, and prognostic factors. CGGA database (http://www.cgga.org.cn/index.jsp) was used to retrieve the mRNA sequencing data of 1018 glioma samples. Since glioma has no normal tissue data in TCGA, to analyze differential gene expression, we combined 1152 cases of normal brain tissue in the GTEx (https://xenabrowser.net/datapages/) database. Using the annotation platform, the Ensembl Gene IDs were transformed into matching gene symbols. The mean value was used if more than one probe targeted the same Entrez Gene ID. In addition, clinical data for each CGGA sample was retrieved from the CGGA database (21). The GTEx and TCGA databases were transformed into log2 (FPKM+1), and the CGGA database was transformed into log2 (RNA-seq+1). Previous research found 309 genes involved in fatty acid metabolism (22–24). In TCGA cohorts, 191 candidate genes were chosen among these genes.

### Enrichment Analysis of DEGs

Fatty acid metabolism-dependent DEGs in normal and tumor tissues were used to evaluate the R package “limma”. Statistical significance was assigned to genes with an FDR of less than 0.05. To validate the key biological properties and cellular functional pathways connected to fatty acids, “cluster profile” software was used to conduct a GO and KEGG enrichment analysis of differential genes. *P* < 0.05 is considered as statistical significance. To show the enrichment analysis findings, we utilized the R software packages “enrich plot” and “ggplot2”.

### Construction of Risk Prediction Model of Fatty Acid Related Genes

We firstly classified the TCGA dataset into a training group and a testing group according to 2:1, while CGGA was used as another validation set. To begin, the expression levels of DEGs associated with fatty acid metabolism were compared to the prognosis findings for each sample. The genes associated with prognosis were selected from the DEGs linked to fatty acid metabolism using univariate and multivariate cox regression methods in the TCGA cohort. Genes having a P value of less than 0.001 were chosen. The mutation and gene association in tumor samples from the training set were analyzed using the R software package “maftools”. The prognosis-related genes were further processed utilizing the “glmnet” R software package, and the prognosis risk score model of glioma OS was constructed using LASSO. The penalty parameter (λ) of the model was determined by ten cross verifications. The risk score for every sample is generated by the method below.


$$Risk\;Score=\sum_{1}^{i}\left(Coefi*ExpGenei\right)$$

“ExpGene” is the transcriptomic value from the predictive risk score model, and “Coef” is the non-zero regression coefficient obtained utilizing LASSO. All samples were separated into two subgroups based on the median value of risk scores: low- and high-risk groups. To examine OS variations between high- and low-score subgroups, Kaplan-Meier (K-M) and the log-rank procedure were performed. The ROC curve was drawn using the R software package “survivalROC” to analyze the predictive risk score model's prediction accuracy. Finally, we checked the test set to see whether the predictive risk score model was reliable.

### PCA of Risk Score Model

We used the limma software package for principal component analysis (PCA) of gene expression to understand the significant differences between the two groups. We first analyzed the face of all DEGs related to fatty acid metabolism by PCA. After that, the gene expression in the predictive risk rating model was analyzed by principal component analysis. Finally, the PCA results are presented on the two-dimensional graph using the “ggplot2” software package.

### The Link Between Risk Ratings and Clinical Characteristics

According to the sample ID, the value of every sample was blended with the associated clinical features. Limma R software tool was adopted to investigate the association between risk scores and clinical data. The TCGA database was also employed to collect the expression levels of immunological checkpoints (symbolized by PD-1/PD-L1 and CTLA4). Immunological checkpoint levels in the two subgroups were then investigated. Simultaneously, clinical data on glioma in the CGGA cohort was gathered to establish the association between clinical factors and risk scores. According to clinical characteristics, the data were separated into two subgroups, and the differences in risk scores were examined. To assess the two groups, the Kruskal Wallis (K-W) method was utilized. A p value of < 0.05 was defined as statistical significance.

### Assessment of Immune Characteristics Between Two Subgroups

Gsva is an unsupervised, nonparametric approach for evaluating route modifications or biological processes using expression matrix samples. The “gsva” R program was employed to assess the differences in biological processes between the lower and higher subgroups. As a reference gene set, we used the “C2. Cp. kegg. V7.4. Symbol” gene set from the molecular feature database (https://www.gsea-msigdb.org/gsea/msigdb). A statistically meaningful enrichment route is one with an FDR of less than 0.05. To calculate the IC50 of temozolomide within every sample, the pRRophetic R program was utilized. The IC50 value represents a substance’s ability to block certain biological or metabolic activities. To estimate the degree of immune-related infiltration in each patient in the TCGA set, ssGSEA was calculated by “GSEABase” and GSVA R packages (25). The data sets were gathered for the prior study’s assessment of immune-related aspects in TME (**Table S1**). The ssGSEA algorithm’s enrichment index reflected the degree to which each immune-related trait was expressed in each sample. The differences in enrichment scores between the lower and higher subgroups were analyzed. The relationship between prognosis-related genes and immune cells was indeed investigated. Finally, in the two risk score subgroups, TIDE was utilized to predict immune checkpoint reaction inhibitors of PD-1 and CTLA-4 (26).

### Construct Nomogram

Nomogram of gender, age, grade, histological type, IDH1 mutation status, pq status, MGMT status, and predictive risk score model was built according to the TCGA cohort, utilizing the “RMS” software package in R to predict the OS of the glioma. To forecast the nomogram’s accuracy, a time-dependent calibration curve was created. Furthermore, a multivariate Cox regression analysis was done to see if a predictive model can be employed as an independent predictor of OS in glioma patients. The AUC was then determined using a ROC curve to confirm the nomogram’s predictive value.

### Analysis of Survival-Related Hub Genes According to the Prediction Model

First, the limma R software program was used to compare the data of two subgroups to identify DEGs, which were defined as genes with an adjusted p-value < 0.05. Based on the string database (https://string-db.org/; version: 11.0), data from the PPI network with interaction scores > 0.40 (median confidence) were produced (**Table S2**). Then, to further analyze and show PPI network data, utilize Cytoscape software (version 3.9.1). Cytohubba is a Cytoscape plug-in that uses a topological technique to find the center gene among all DEGs. Then, the deferentially expressed genes were gathered in normal brain tissue and glioma tissue. The clusterprofiler software tool was employed to do a Go and KEGG enrichment analysis of genes. Finally, a gene from the hub gene was chosen for model validation, and all samples were separated into two subgroups according to this gene’s expression: low expression and high expression. To see whether there was a variation in survival between the two subgroups, Kaplan Meier analysis was utilized. This gene was then investigated for immune cell infiltration.

### Cell Culture and Transfection

The glioma cells (LN229, T98G, U251, U87, and U118) and the normal control cell NHA were cultured in DMEM with 10% FBS at 37°C in a 5% CO2 incubator. The logarithmic phase cells were chosen for the following functional studies. For CCNA2 knockdown or overexpression, glioma cells were transfected with plasmid si-CCNA2 or CCNA2-cDNA utilizing Lipofectamine^®^ 3000 transfection reagent, following the manufacturer’s manual.

### Macrophage Polarization and Co-Culture

To obtain M0 macrophages, THP-1 cells were firstly induced by 320 NM PMA for 24h; To polarize M0 to M2 macrophages, cells were then treated with 20ng/ml IL-4 and 20ng/ml IL-13 for 48h. M0 macrophages were put into upper wells for cell co-culture, and U251-NC and U251-shCCNA2 cells were seeded into the bottom well. Macrophages were then collected and labeled with M1- and M2-like markers to identify the polarization features.

### qRT-PCR

Total RNA was extracted from cells utilizing Trizol reagent (Invitrogen, America), then cDNAs were created using the HiScript Synthesis kit (Vazyme, China). Subsequently, the PCR mixture consisted of cDNA, ddH2O, primer, and SYBR Green Master Mix. Finally, qRT-PCR amplification was measured on the StepOnePlus Real-Time PCR system (Applied Biosystems, US). Primers were as follows: CCNA2, forward-5’- TGG AAA GCA AAC AGT AAA CAG CC-3’, reverse-5’- GGG CAT CTT CAC GCT CTA TTT-3’; iNOS, forward-5’- TCA TCC GCT ATG CTG GCT AC-3’, reverse-5’- CCC GAA ACC ACT CGT ATT TGG-3’, TNF-α, forward-5’-GAG GCC AAG CCC TGG TAT G-3’, reverse-5’-CGG GCC GAT TGA TCT CAG C-3’; IL-1β, forward-5’- GAA ATG CCA CCT TTT GAC AGT G-3’, reverse-5’-TGG ATG CTC TCA TCA GGA CAG-3’, CD206, forward-5’- CTA CAA GGG ATC GGG TTT ATG GA-3’, reverse-5’-TTG GCA TTG CCT AGT AGC GTA-3’, Arg1, forward-5’- TTG GGT GGA TGC TCA CAC TG-3’, reverse-5’-GTA CAC GAT GTC TTT GGC AGA-3’; YM1/2, forward-5’-TCA GCA GGT TCC CTA CGC A-3’, reverse-5’-GCA GGA TTT GCC AGT GAA GTC-3’.

### Western Blot

The western blot procedure was conducted as described previously (27). Primary antibodies in this study included iNOS (1:1000, Proteintech), CD206 (1:1000, Proteintech), β-Actin (1:1000, Bioss).

### Flow Cytometry

To examine the polarization of macrophages, M0 macrophages were collected, fixed, then incubated with F4/80-FITC, iNOS-APC, and CD206-PE for 30 min at four °C, based on the manufacturer’s instructions. Cells were determined using a flow cytometer (Cytoflex, USA), and the data were analyzed using FlowJoTM software (Version 10.7.1).

### CCK-8 and Clone Assay

CCK-8 and plate colony assays were utilized to examine the glioma cells’ proliferation capacity. The CCK8 kit (Beyotime, China) was used to quantify cell growth at 0h, 24h, 48h, 72h, and 96h by the manufacturer’s protocol. Absorbance at 450 nm was determined on enzyme labeling (Thermo, USA). For the cloning test, about 500 cells from various groups were placed in each well of a six-well plate. Once colonies appeared, 4% paraformaldehyde and crystal violet were employed to stain and fix cells.

### Transwell Invasion Assay

After si-CCNA2 and CCNA2-cDNA transfection, 5×104 glioma cells were placed into the upper chambers of Transwell in an empty DMEM medium, which was pre-coated with Matrigel (Biosciences, USA), while the lower chamber was filled with DMEM containing 10% FBS. For macrophage migration assay, M2 macrophage was placed on upper chambers, and glioma cells were added to the lower chamber. After 24 hours, the invasive cells were fixed with 4% formalin, then stained with 0.1% crystal violet. The stained cells were visualized and counted in an inverted microscope.

### Subcutaneous Xenograft Assay

Nanjing medical university animal care and use committee and followed guidelines for animal welfare. Four-week-old BALB/c male nude mice were used to construct the xenograft model, which randomly classified into two groups. The glioma cells infected with siCCNA2 or control were subcutaneously embedded in nude mice, respectively. Tumor volumes were measured every one week. After one month, mice were euthanatized, and subcutaneous tumors were removed. Tumor weight and pictures were finally recorded.

### Statistical Analysis

To assess the differences between poor and good score subgroups, the Wilcoxon rank sum test was utilized. The variation in OS between the good and poor subgroups was determined using Kaplan Meier analysis. The independent determinants of OS in glioma were determined using the Cox regression procedure. The predictive efficacy of the nomogram, clinical factors, and predictive risk score model was evaluated using a ROC curve. R 4.0.4 was used to conduct all statistical analyses (*P* < 0.05).

## Results

### Differential Expression Analysis Between Normal and Glioma Samples

We analyzed the transcriptional activity of FAMDs in tumor and normal samples and identified 191 genes in TCGA datasets with an FDR < 0.05. It comprises 95 down-regulated genes and 96 genes that have been up-regulated (**Figure S1A**). Then, to double-check, we performed KEGG and GO enrichment analysis. Fatty acid metabolism, catabolism, and other activities have been discovered to be substantially abundant in biological processes (**Figure S1B**). KEGG terms for fatty acid breakdown, metabolism, and biosynthesis are abundant (**Figure S1C**).

### Establishment of the Prognostic Model in the Training Set

**Figure 2:**
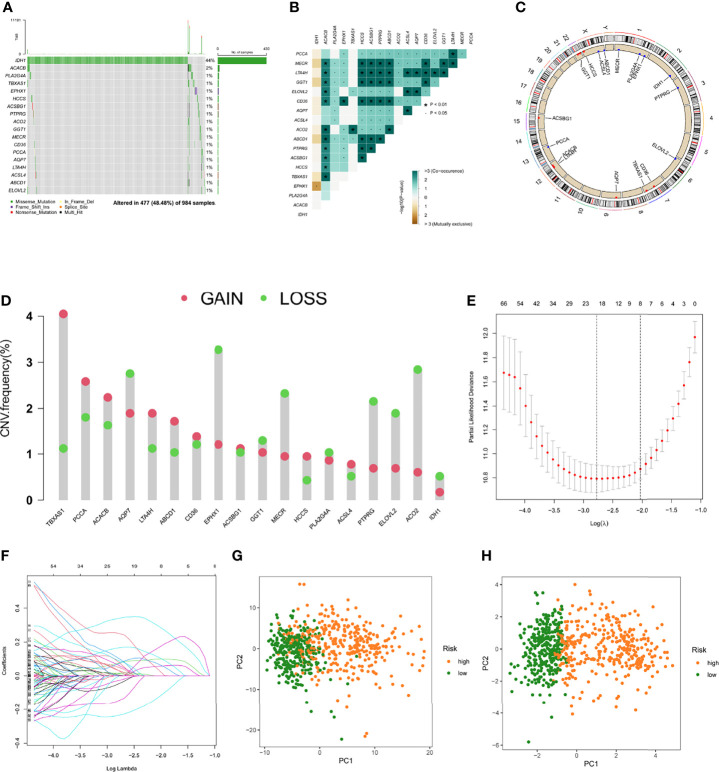
Predictive risk score model development. **(A)** The frequency of mutations in 18 FAMDs in 984 glioma patients from the TCGA cohort. **(B)** Mutation co-occurrence and selection analysis for 18 genes involved in fatty acid metabolism. Green denotes co-occurrence; purple denotes exclusion. **(C)** Locations of CNV alterations in FAMDs on 23 chromosomes. **(D)** Frequencies of CNV gain, loss, and non-CNV among FAMDs. **(E)** LASSO coefficients for the FAMDs involved in fatty acid metabolism. **(F)** Gene discovery for the construction of a predictive risk score model. **(G)** Principal component analysis of FAMDs in glioma. **(H)** In the TCGA cohort, principal component analysis was used to identify cancers from standard samples using a fatty acid metabolism vulnerability index. High-risk patients were represented by the red group, whereas the blue group represented low-risk patients. *P<0.01, •P<0.05.

Glioma patients in the TCGA database were randomly divided into a training cohort and a testing cohort at a 2:1 ratio, we used the training set to construct a prognostic model. On 191 FAMDs, a Univariate Cox regression analysis was conducted. With a p value of 0.001, a total of 133 genes associated with prognosis were discovered (**Table S3**). The somatic mutation profile of the 18 genes involved in fatty acid metabolism that have been linked to prognosis was first summarized. As indicated in **Figure 2A**, a total of 477 of 984 glioma samples had mutations in FAMDs, with a prevalence of 48.48 percent. IDH1 has the most mutations. Further research revealed that ACACB and TBXAS1 had a mutation co-occurrence association (**Figures 2B–D**). The number of genes was then reduced using the LASSO Cox regression analysis. Finally, ten genes were employed to create a predictive risk score model (ACO2, PTGR1, GPD1, HCCS, ABCD1, RETSAT, SMS, CA2, ELOVL5, and SCD) (**Figures 2E, 2F**). The following formula was used to compute every sample’s score: risk score = (-0.362) × ACO2 + (0.246) × PTGR1 + (0.127) × GPD1 + (0.411) ×HCCS + (0.241) × ABCD1 + (0.326) × RETSAT + (0.338) × SMS + (0.202) × CA2 + (0.256) × ELOVL5 + (-0.429) × SCD. **Table S4** shows how this was accomplished. To fully differentiate glioma samples, the risk score model was applied (**Figures 2G, 2H**)

**Figure 3:**
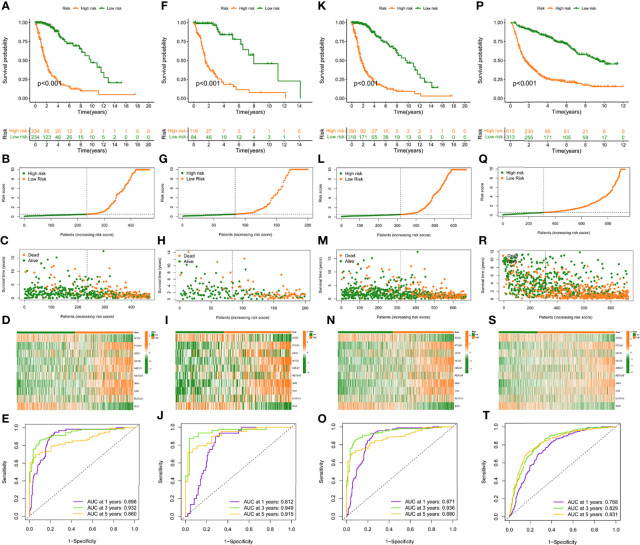
Construction and validation of risk model in TCGA training, testing, entire cohort, and CGGA cohort. **(A, F, K, P)**, **(K–M)** curves of subgroups for OS in TCGA training, testing, entire cohort, and CGGA cohort. The distribution plots of risk score **(B**, **G**, **L**, **Q)**, survival status **(C**, **H**, **M**, **R)**, and 10 selected FAMDs **(D**, **I**, **N**, **S)** in TCGA training, testing, entire cohort, and CGGA cohort. **(E**, **J**, **O**, **T)** ROC curve analysis of risk score in predicting 1-, 3-, and 5-year OS in TCGA training, testing, entire cohort, and CGGA cohort.

In the training set, patients were classified into the high- and low-risk group according to the median value. K-M curves showed that the high-risk group had a poor prognosis compared to low-risk patients (p < 0.05) (**Figure 3A**). The highest AUC value reached 0.898 for 1-year OS, and 0.932 for 3-year OS (**Figure 3E**). To better discern the survival differences between these two groups, we ranked all patients based on their risk scores, then plotted to scatter maps to describe each patient’s survival time and status (**Figures 3B–D**).

### Validation of the Prognostic Model in the Testing Set, Entire TCGA and CGGA Set

To verify the robustness of this risk model, the predictive performance was determined in the testing set, entire TCGA and CGGA set. Similarly, patients in these cohorts were categorized into high- and low-risk groups, according to the cut-off value achieved in the training cohort. There were 116 high-risk patients, 84 low-risk patients in the testing set, and 350 high-risk patients, 318 low-risk patients in the entire TCGA set, and 615 high-risk patients, 313 low-risk patients in the CGGA set.

K-M curves indicated that high-risk individuals in these cohorts had a shorter OS than the low-risk group (p < 0.05) (**Figures 3F, K, P**). The AUC for predictive OS was 0.812 at 1 year, 0.949 at 3 years in the testing cohort, 0.871 at 1 year, 0.936 at 3 years in the entire TCGA cohort, 0.768 at 1 year, 0.829 at 3 years in the CGGA cohort (**Figures 3J, O, T**). Also, we graded respectively different populations in these cohorts based on their risk score. The high-risk group exhibited a poor prognosis compared to low-risk patients, which was consistent with the findings in the training set (**Figures 3G–I, L–N, Q–S**).

### The Connection Between the Risk Model and Clinical Information

**Figure 4:**
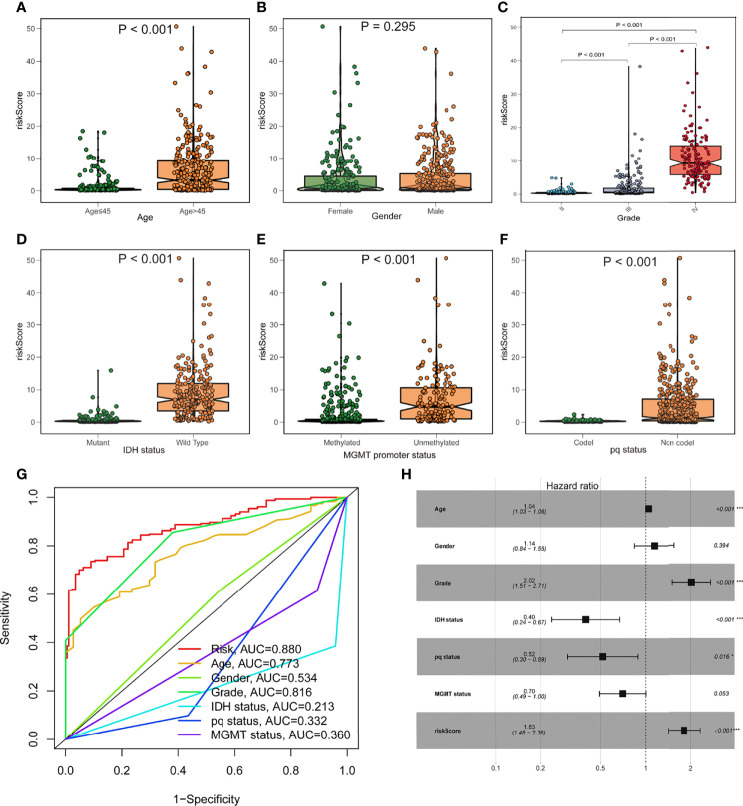
Prognostic value of the fatty acid metabolism score model in glioma patient survival. **(A–F)** The association between risk score and clinicopathological variables such as age **(A)**, gender **(B)**, grade **(C)**, IDH1 status **(D)**, MGMT promoter status **(E)**, and pq status **(F)**. **(G)** ROC curves are used to assess the predictive power of clinical characteristics. **(H)** The forest plot of the TCGA cohort’s multivariate Cox regression analysis. *p < 0.05; ***p < 0.001.

To investigate the relationship between the risk model and clinical features, we determined the risk scores of related samples in terms of age, gender, grade, IDH1 mutation status, pq status, and MGMT status. Although there was no significant difference in risk score by gender, significant variations in risk score by age, grade, IDH1 mutation status, pq status, and MGMT status (p<0.05; **Figures 4A–F**). Higher scores were linked to being older, having higher grades, wild type IDH1 mutations, unmethylated MGMT status, and non-codel pq status. The accuracy of the prognostic risk model was validated by plotting a time-dependent ROC at 5 years (**Figure 4G**). Age, grade, IDH status, pq status, and risk score were predictive factors of OS in multivariate analysis among characteristics linked with OS in univariate analysis (**Figure 4H**).

### Construction of Survival-Prediction Nomogram

**Figure 5:**
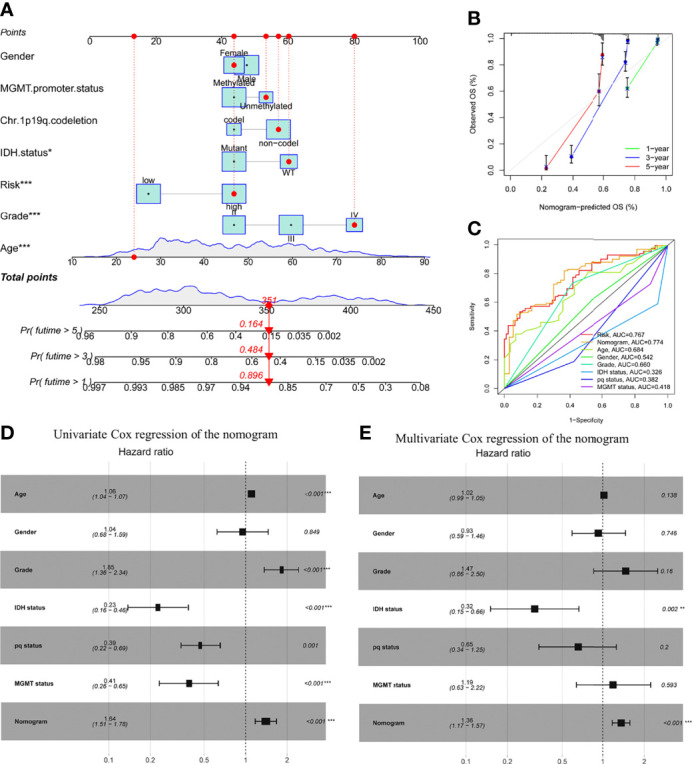
Prognostic efficacy of risk score in OS of patients from the TCGA cohort when combined with clinical-pathological features. **(A)** Nomogram indicates OS in the TCGA cohort of patients. **(B)** The nomogram’s calibration plots. The y-axis represents actual survival, whereas the x-axis represents nomogram-predicted survival. **(C)** Receiver operating characteristic charts for risk score and clinical features. **(D, E)** The nomogram’s univariate and multivariate Cox regression analyses. *p < 0.05; **p < 0.01; ***p < 0.001..

A nomogram comprising gender, age, grade, IDH1 mutation, MGMT status, pq status, and risk score was created to predict OS in glioma tissues (**Figure 5A**). **Figure 5B** shows calibration curves for patients with gliomas at 1, 3, and 5 years, demonstrating that nomograms may reliably predict OS in these patients. The nomogram (AUC = 0.774) has a stronger predictive value than a single indicator such age (AUC = 0.684), grade (AUC = 0.660), or the prognostic risk scoring model (AUC = 0.767; **Figure 5C**). The predictive risk score model and grade were shown to be independent prognostic factors in multivariate and univariate Cox regression analysis (**Figures 5D, E**).

### Response of Two Groups to Crizotinib

**Figure 6:**
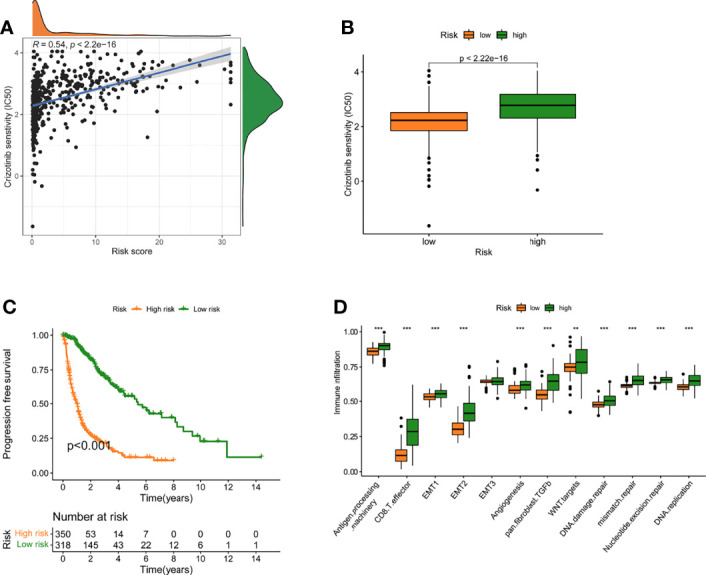
A model of fatty acid metabolism in the context of chemotherapy. **(A)** The relationship between patient risk ratings and crizotinib’s projected IC50 value. **(B)**The disparities in crizotinib response between groups with poor and good risk scores. **(C)** The TCGA cohort’s progression-free survival (PFS) was compared between low- and high-risk score groups. **(D)** Variation in stroma-activated networks between groups with low and high-risk scores (**p < 0.01; ***p < 0.001).

The association between risk level and medication resistance was examined since risk score is linked to poor prognosis. The “pRRophic” R package has been used to estimate the therapeutic efficacy of crizotinib in the TCGA cohort using the half maximal inhibitory concentration (IC50). For the management of glioma, samples with a low-risk score were more susceptible to crizotinib (**Figures 6A, B**). In TCGA data, there was a high association between chemotherapy drugs sensitivity and risk score at 3 years, according to PFS (progression-free survival) (**Figure 6C**). The elevated score was significantly closely linked with the intensive activation of matrix pathways, including epithelial mesenchymal transformation 1 (EMT1), angiogenesis, and Wnt targets, according to an analysis of the activity of matrix-related pathways leading to chemotherapy resistance (**Figure 6D**).

### GSVA

**Figure 7:**
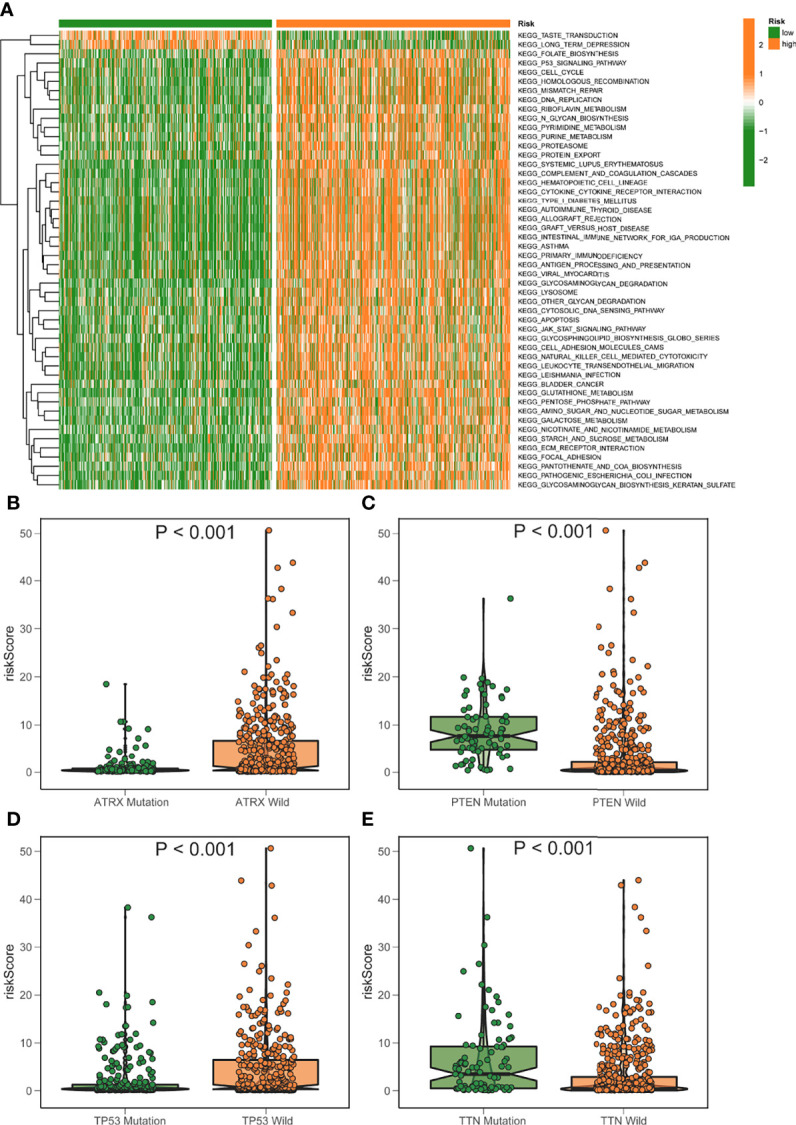
GSVA analysis of elevated/low score groups diagram. **(A)** GSVA enrichment heatmap for excellent and poor score groups. **(B–E)** Varied mutant genes, namely ATRX mutation **(B)** PTEN mutation **(C)** TP53 mutation **(D)** and TTN mutation **(E)** have different fatty metabolism scores.

Gsva enrichment analysis showed that most metabolic pathways, including the biosynthetic metabolic pathway of unsaturated fatty acids, increased in the low-risk score. Genetic abdominal muscle analysis of high scoring population showed that it was related to immunity, such as primary immunodeficiency, natural killer cell mediated cytotoxicity, etc. (**Figure 7A**). The risk score was negatively connected with the fatty acid metabolism score, which was generated using ssGSEA, which examined the expression of fat metabolism-related genes in glioma patients and was consistent with GSVA (**Figures S2A–C**). Although the fat metabolic score could reliably predict the survival in patients at 5 years using a time-dependent ROC, the predictive value (AUC = 0.385) was poorer than the risk rating model (AUC = 0.880) (**Figures S2D–E**). In addition, we compared several mutant genes with high mutation frequency in glioma, TP53, ATRX, PTEN, and TTN. Patients with TP53 and ATRX mutations had higher risk scores and statistically significant differences (**Figures 7B–E**).

### Characteristics of the Immune System in the High- and Low-Risk Groups

**Figure 8:**
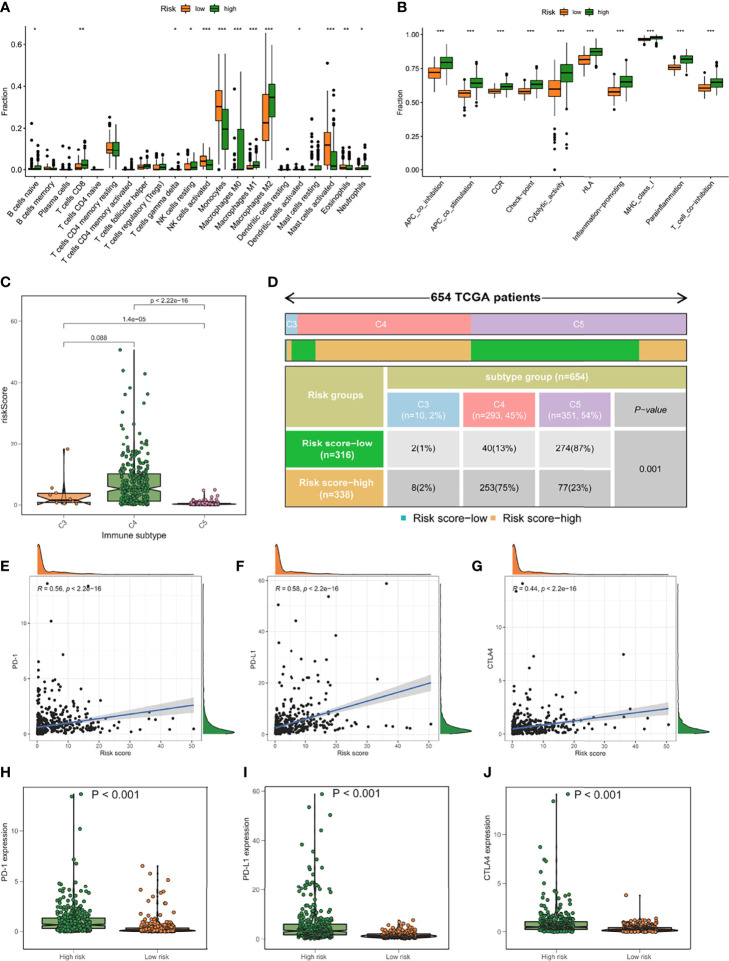
Model of fatty acid metabolism in immunotherapy. **(A)** The difference in immune infiltration between high-risk and low-risk scores. **(B)** The difference between subjects with a high score and those with a poor rating in terms of the known function related to immune modulation. **(C, D)** The distribution of immunological subtypes (C3, C4, and C5) across risk groupings are shown in a heatmap and table. **(E–G)** Immune checkpoint transcriptional activation and hazard score, PD-L1**(E)**, PD1**(F)**, and CTLA3 correlation analysis **(G)**. **(H–J)** The disparities between the low-risk and high-risk groups in terms of identified Immune checkpoint genes. *P<0.05, **P<0.01, ***P<0.001.

The high score group demonstrated an increasing trend in T cells CD8, macrophage M1, and macrophage M2 compared to the low score group, consistent with the high score group’s reduced survival rate. The group with the lowest ranking had a higher increase in monocytes and mast cells activated (**Figure 8A**). Furthermore, the high-risk group’s type I IFN response, type II IFN response, checkpoint, HLA, T cell co-inhibition, T cell co-stimulation, etc., were all activated, suggesting that patients with immune suppression in the high-risk group would react to immunotherapy (**Figure 8B**). The TCGA samples were additionally immunotype, and the elevated/low score groups had substantial variations in C3, C4, and C5 (**Figures 8C, D**). CTLA4 and PD-1 inhibition are two examples of immunotherapy that have achieved significant advances in cancer treatment. As a result, we looked at the predictive risk score model’s capacity to distinguish between individuals who react to immune checkpoint inhibitor treatment in various ways. The findings revealed that the projected risk score was highly connected with immune checkpoint gene expression (PD-1, PD-L1, and CTLA4) and that the expression was considerably higher in the high-risk group. This shows that our risk ratings for fatty acid metabolism might be relevant in determining immunotherapy prognosis (**Figures 8E–J**).

### Verification of Prediction Model by the Hub Gene in DEGs of High- and Low-Risk Groups

**Figure 9:**
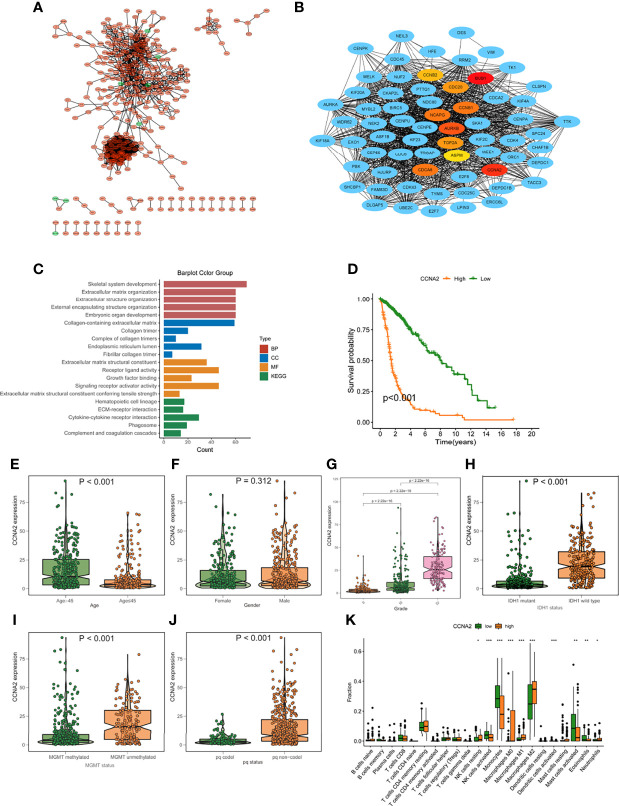
Protein-protein interaction (PPI) graph. **(A)** PPI network generated by Cytoscape (red): DEGs that exhibited strongly in the excellent score category; green: DEGs that showed highly in the low-risk score group. **(B)** The top ten hub genes were chosen by cytoHubba. **(C)** The findings of GO and KEGG enrichment analysis on DEGs. **(D)** Mortality analysis for patients divided into subgroups based on CCNA2 mRNA expression. **(E-J)** The difference in CCNA2 mRNA expression between various clinical characteristics, such as age **(E)**, gender **(F)**, grade **(G)**, IDH1 mutation **(H)**, MGMT promoter status **(I)**, and pq status **(J, K)** In subjects with elevated/low CCNB1 mRNA expression, the number of TME-infiltrating cells. *p < 0.05; **p < 0.01; ***p < 0.001.

The expression patterns of DEGs in the low- and high-risk groups were analyzed using the string online database. As illustrated in **Figure S3**, a protein interaction diagram of DEGs was created. PPI network data is processed and shown using the Cytoscape program. **Figure 9A** depicts the DEG interaction, with red indicating up-regulated transcripts in the excellent score group and green indicating up-regulated genes in the poor score group. Cytohubba is a Cytoscape plug-in that helps you find the core gene among DEGs. As seen in **Figure 9B**, we chose 10 genes from the network. To further understand the roles of DEGs, researchers used the R software tool “goplot” to conduct a Go and KEGG study. The genes are engaged in skeletal system development, extracellular matrix organization, extracellular structure organization, etc., according to GO findings. These genes were shown to be abundant in hematopoietic cell lineage, ECM-receptor interaction, cytokine-cytokine receptor interaction, etc., according to KEGG findings (**Figure 9C**). For verification, we used CCNA2 in the hub gene. The expression levels of CCNA2 were shown to be substantially linked with the mortality of glioma patients in a survival study (**Figure 9D**). Furthermore, CCNA2 expression increased with age and grade. However, in the IDH1 mutation, pq codel, MGMT methylated group, CCNA2 expression was dramatically reduced (**Figures 9E–J**). To see whether there is a difference in TME immune infiltration between individuals with high- and low– CCNA2 expression. Compared to patients with low transcription, tumors with high CCNA2 expression showed considerably higher macrophages M2 and M1 (**Figure 9K**).

### CCNA2 Promoted Malignant Glioma Progression *In Vitro*, and *Vivo*

**Figure 10:**
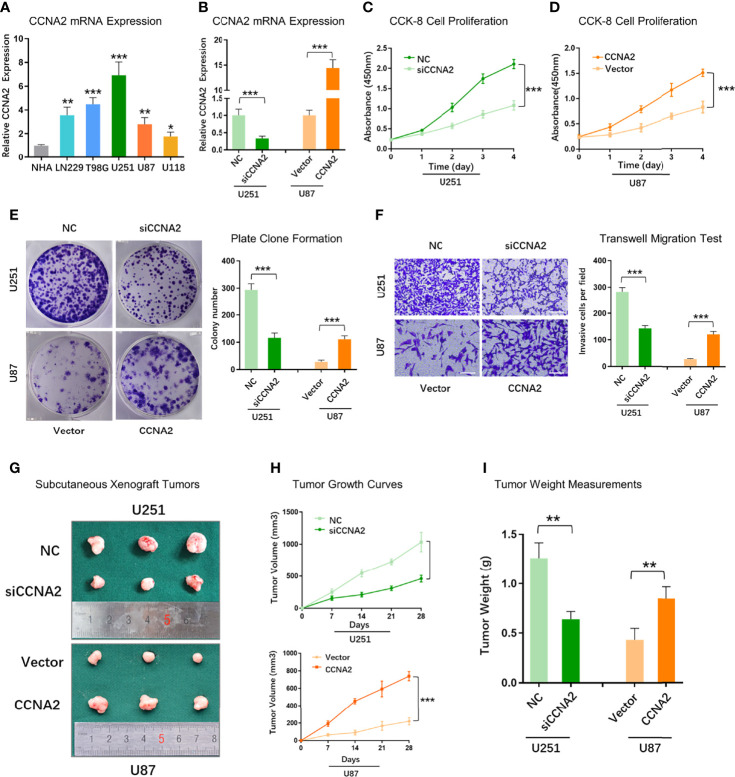
CCNA2 promoted glioma cell proliferation and migration. **(A)** qRT-PCR of CCNA2 mRNA expression in NHA and glioma cells. **(B)** qRT-PCR of CCNA2 levels in U251 and U87 cells transfected with si-CCNA2 and CCNA2-cDNA. **(C, D)** CCK-8 of the cell growth curve in transfected U251 and U87 cells. **(E)** Colony assay in transfected U251 and U87 cells. **(F)** Transwell invasion test in transfected U251 and U87 cells. **(G)** Representative images of subcutaneous xenograft tumors implanted with U251-siCCNA2 and U87-CCNA2 cells. **(H, I)** Tumor volume and tumor weight of indicated xenograft models. *P< 0.05, **P < 0.01, ***P < 0.001.

To further validate the role of CCNA2 in glioma, we assessed if changes in CCNA2 expression affect glioma cell proliferation and malignant abilities. We first detected the expression of CCNA2 in different cells by qRT-PCR and found that CCNA2 mRNA levels were significantly high in LN229, T98G, U251, U87, and U118 cells compared to NHA (**Figure 10A**). We chose U251 for si‐CCNA2 and U87 for CCNA2 cDNA transfections. The results indicated that CCNA2 shRNA reduced CCNA2 expression in U251 cells, whereas CCNA2 cDNA up‐regulated CCNA2 expression in U87 cells (**Figure 10B**). The CCK-8 (**Figures 10C, D**) and colony formation (**Figure 10E**) results demonstrated that down‐regulation of CCNA2 expression inhibited U251 cell proliferation, whereas up‐regulation of CCNA2 expression promoted tumor U87 cell proliferation. In transwell assay, suppressing CCNA2 expression significantly resulted in a reduction of invaded U251 cells, whilst upregulating CCNA2 expression significantly expanded the amount of invaded U87 cells (**Figure 10F**). Moreover, the animal experiment suggested that CCNA2 knockdown suppressed glioma growth *in vivo*, while CCNA2 overexpression promoted tumor growth (**Figures 10G–I**). These results indicated that CCNA2 facilitated *in vitro* and *in vivo* proliferation, migration, and invasion of glioma cells.

### CCNA2 Regulated Macrophages Polarization

**Figure 11:**
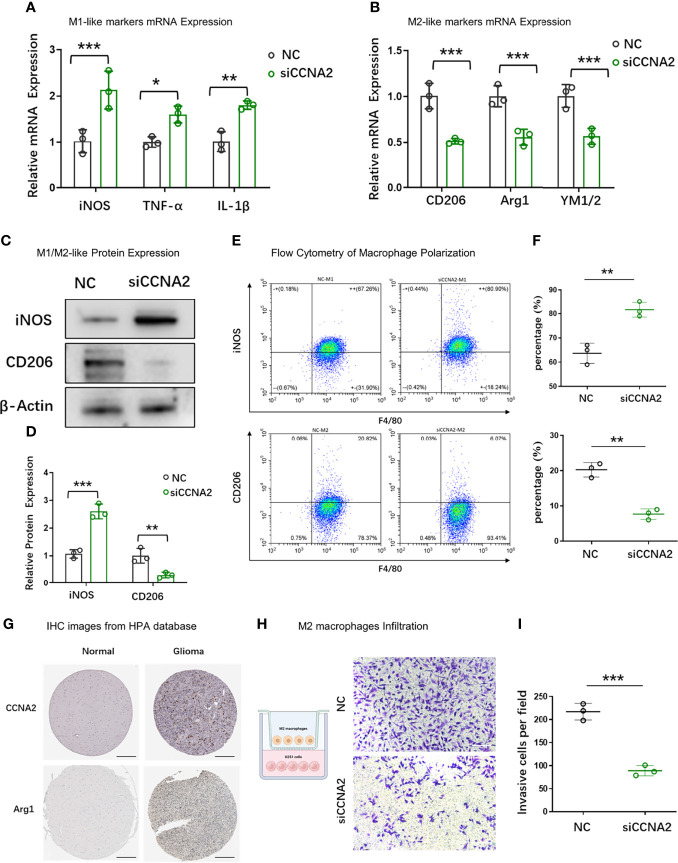
Effect of CCNA2 on macrophage polarization. **(A, B)** mRNA expression levels of M1-like and M2-like markers of macrophages after co-cultured with U251-siCCNA2 cells and controls. **(C, D)** Protein levels of iNOS and CD206 of macrophages after co-cultured with transfected cells. **(E, F)** Flow cytometry analysis of iNOS and CD206 expression in indicated groups. **(G)** Representative IHC images of CCNA2 and M2-like marker Arg1 in normal brain and glioma tissues from the HPA database. **(H, I)** M2 macrophage infiltration after co-cultured with indicated cells. *P < 0.05, **P < 0.01, ***P < 0.001.

To further examine the relationship between CCNA2 and macrophage polarization *in vitro*, we chose U251 cells for further study. U251 cells were transfected with shCtrl and shCCNA2, then co-cultured with macrophages. The result of qRT-PCR showed that the macrophages co-cultured with U251-siCCNA2 had higher M1-like markers iNOS, TNF-α, and IL-1β (**Figure 11A**), while having lower M2-like markers CD206, Arg1 and YM1/2 compared with controls (**Figure 11B**). Western blot and flow cytometry showed that knockdown of CCNA2 significantly inhibited the shift to M2 macrophages compared with the NC group (**Figures 11C–F**). IHC staining indicated that CCNA2 expression and M2 macrophage marker CD206 were stronger in GBM than usual (**Figure 11G**). Transwell assay showed that silencing CCNA2 in U251 cells significantly decreased the M2 macrophage infiltration (**Figures 11H, I**). The results showed that CCNA2 regulated the polarization of macrophages.

## Discussion

For a long time, people have been considering the metabolic conversion of cancer cells from the perspective of how and why cancer cells give priority to the use of glucose through aerobic glycolysis (the so-called Warburg effect). In the past few years, we have made great progress in understanding the newly connected metabolic networks intertwined with carcinogenic signals of cancer cells. Among these metabolic reprogramming theories, the deregulation of lipid metabolism is considered one of the primary metabolic markers of tumor cells (28). Aside from aberrant glucose metabolism, cancer cells’ lipid, nucleic acid, and amino acid metabolism will vary (29). It has been discovered that lipid initiating changes plays a crucial role in membrane synthesis, signal transduction, and energy production of cancer cells (30). Most research now concentrates on the involvement of a single gene in glioma, and the full impact of numerous fatty acid metabolism genes is unknown. Understanding the significance of distinct fatty acid metabolism processes in glioma and their association with immunotherapy may suggest appropriate treatment methods by increasing the knowledge of fatty acid metabolism in tumor progression.

There are many successfully constructed prognostic models for glioma, such as the methylation-related or immune-related models in glioma (31–35) and other tumors (36, 37). But research focused on fatty acid metabolism. The connection between FAMDs and glioma was investigated. Univariate Cox regression assessment and lasso cox regression analysis were employed to assess prognostic risk. In the TCGA training cohort, a scoring model was developed for 191 expression levels associated with metabolism in normal and tumor brain tissue. In patients with glioma, the predictive risk score approach was utilized to predict OS. And we validate the model in the TCGA testing cohort, TCGA entire cohort, and CGGA cohort. We used a clinical correlation study to better know about the importance of these genes in glioma. The poor score subgroup and the good score subgroup had different survival rates. The testing cohort yielded the same findings, showing that the predictive risk score model may identify patients with low-risk viability. The predicted risk score model was developed using a multivariate analysis of prognostic data. Furthermore, by combining nomograms with specific clinicopathological variables, we may improve the prediction power of our prognostic risk score model.

Because gliomas need to be treated with a combination of chemotherapy drugs in the later stage. As a result, to better understand the relevance of the predictive risk model in glioma, we evaluated the medication treatment responses of patients in the good and poor score groups. The risk score was positively correlated with crizotinib resistance, which was consistent with previous studies (38). Our personalized PFG score for patients with glioma can be used for the prognosis of glioma. Individuals with elevated ratings had considerable matrix activation, suggesting the existence of chemoresistance, which was similar to previous findings. Chemoresistance is common in individuals with high hazard scores, hence immunotherapy is usually avoided. Most individuals with poor glioma are not candidates for immunotherapy (Immunologic checkpoint, inhibiting [PD-1/L1 and CTLA4]). As a result, distinguishing people who are candidates for immunotherapy is critical in clinical practice. Patients with high-level scores are rich in inhibitory immune cells and immune inflammatory cells. In addition, patients with better scores have the functions of activating type I and II IFN responses which can promote inflammation. All of this suggests that individuals with good scores are candidates for immunotherapy, which is consistent with cancer immune dysfunction and resistance being predicted.

Because the high- and low-score subgroups varied significantly, the distinct genes in the 2 categories were investigated further. CCNA2 was discovered to be necessary. Not only was CCNA2 mRNA expression linked to the clinical stage, but it was also linked to a worse prognosis. The CCNA2 knockdown has also been shown to suppress cell growth by impairing cell cycle progression and inducing cell apoptosis (39). Rui et al. reported that the ability of cancer cell proliferation, invasion, and metastasis was decreased after down-regulated expression of CCNA2 in prostate cancer cell lines (40). However, no research on CCNA2 in glioma has been published, and the process in glioma has to be investigated further.

In conclusion, the fatty acid predictive risk score model may be utilized to assess the fatty acid metabolism network completely. The calculated risk score may be used to classify a patient’s clinicopathological characteristics, such as clinical stages. Furthermore, the risk score is linked to the prognosis of patients and may predict immunotherapy. As a result, the clinical practice may be successfully guided by relative risk and clinical-stage to produce a more tailored clinical follow-up approach. These results present a unique, efficient, and accurate predictive and immunotherapy response prediction methodology, paving the way for tailored cancer immunotherapy in the future.

## Data Availability Statement

Public data in this study could be found as follows: TCGA (https://portal.gdc.cancer.gov/) and CGGA database (http://www.cgga.org.cn/index.jsp), normal brain tissue from GTEx database (https://xenabrowser.net/datapages/).

## Ethics Statement

The animal study was reviewed and approved by Ethics Committee of the Nanjing Medical University (No. IACUC-2012013).

## Author Contributions

FJ and YH conceived the manuscript. NZ, YM, and XT wrote the manuscript. JW, FL, and CW conducted the statistical analysis. All authors reviewed the paper and approved the submission.

## Funding

This study was funded by the National Natural Science Foundation of China (NO.8172539) and the Nanjing Municipal Science and Technology Bureau (Grant number 2019060002). The funding bodies had no role in the study design, data collection, analysis, and interpretation of data.

## Conflict of Interest

The authors declare that the research was conducted in the absence of any commercial or financial relationships that could be construed as a potential conflict of interest.

## Publisher’s Note

All claims expressed in this article are solely those of the authors and do not necessarily represent those of their affiliated organizations, or those of the publisher, the editors and the reviewers. Any product that may be evaluated in this article, or claim that may be made by its manufacturer, is not guaranteed or endorsed by the publisher.

## Supplementary Material

Supplementary Figure 1 Differential expression analysis between normal and glioma samples.

Supplementary Figure 2 Fatty acid metabolism score.

Supplementary Figure 3 The protein interaction diagram.

Supplementary Table 1 The gene collections were gathered for the prior study’s assessment of immune-related aspects in TME.

Supplementary Table 2 PPI network data.

Supplementary Table 3 133 genes associated with prognosis after univariate cox analysis.

Supplementary Table 4 10 genes were utilized to create a predictive risk score model.
