# Supplementary figures and images for: Characterization of Fatty Acid Metabolism-Related Genes Landscape for Predicting Prognosis and Aiding Immunotherapy in Glioma Patients
Source: Front Immunol. 2022 Jul 12;13:902143. doi: 10.3389/fimmu.2022.902143 (PMC9315048; doi:10.3389/fimmu.2022.902143)

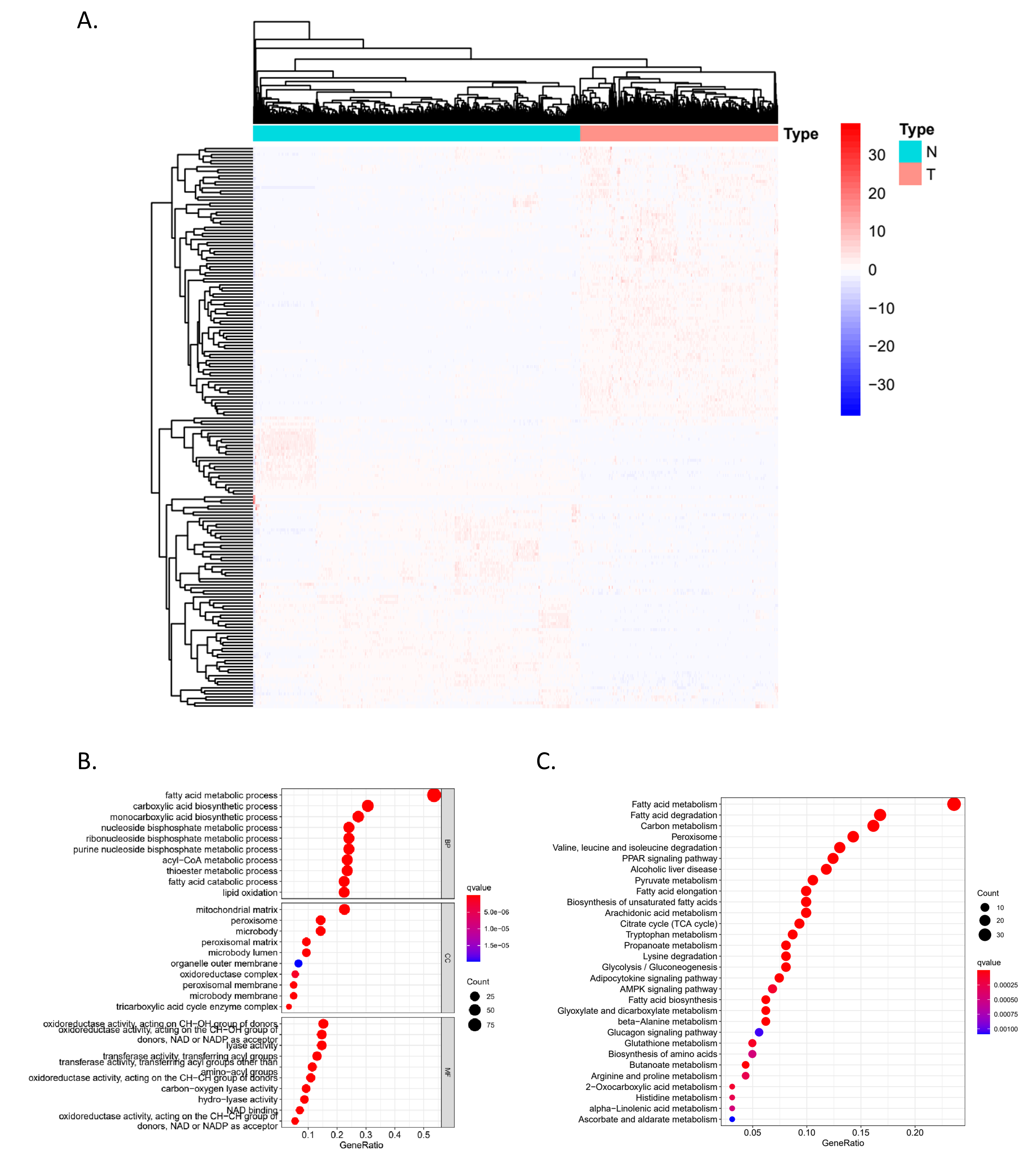

Supplement: Supplementary Figure 1 — Differential expression analysis between normal and glioma samples. [file Image_1.tif]

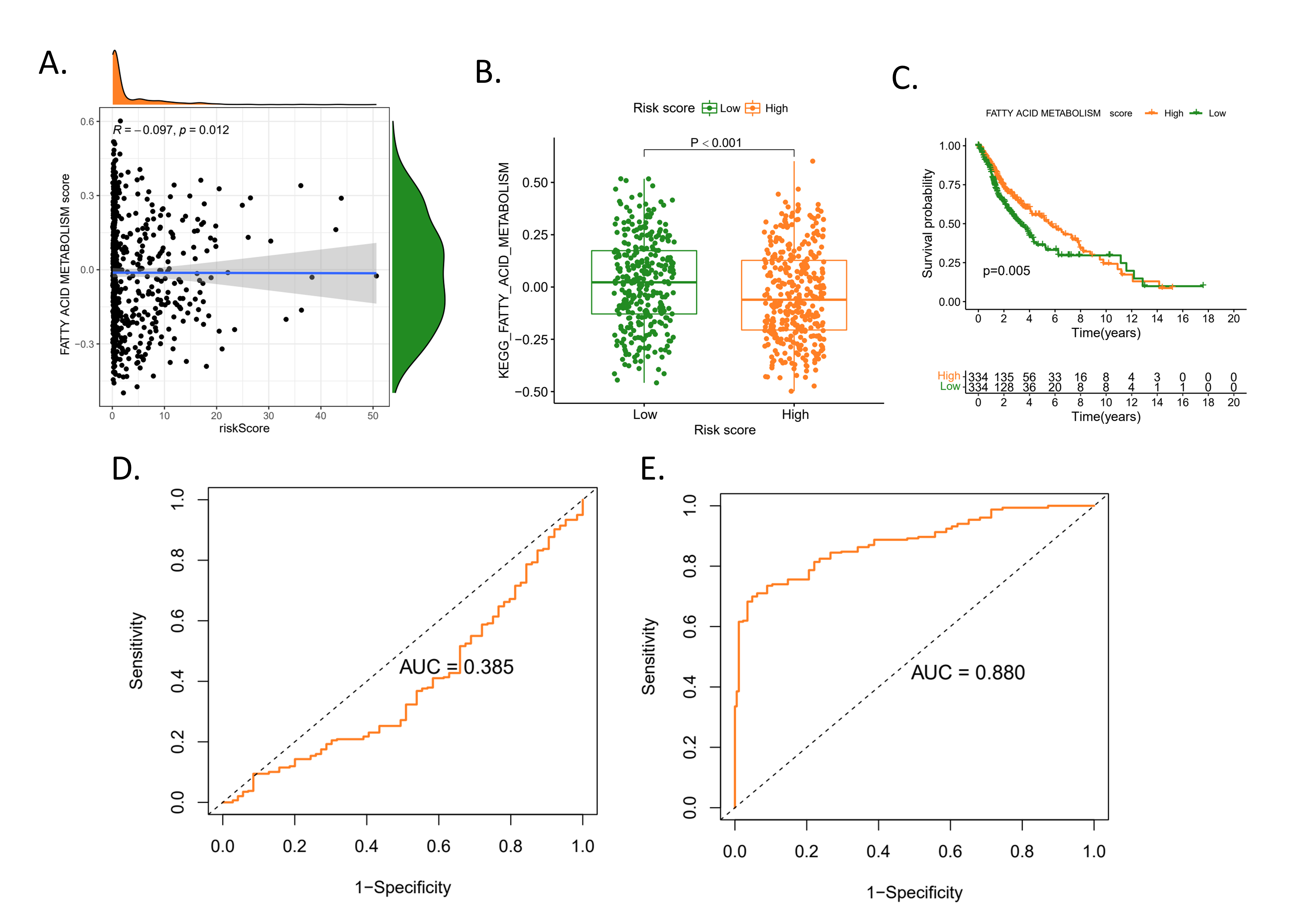

Supplement: Supplementary Figure 2 — Fatty acid metabolism score. [file Image_2.tif]

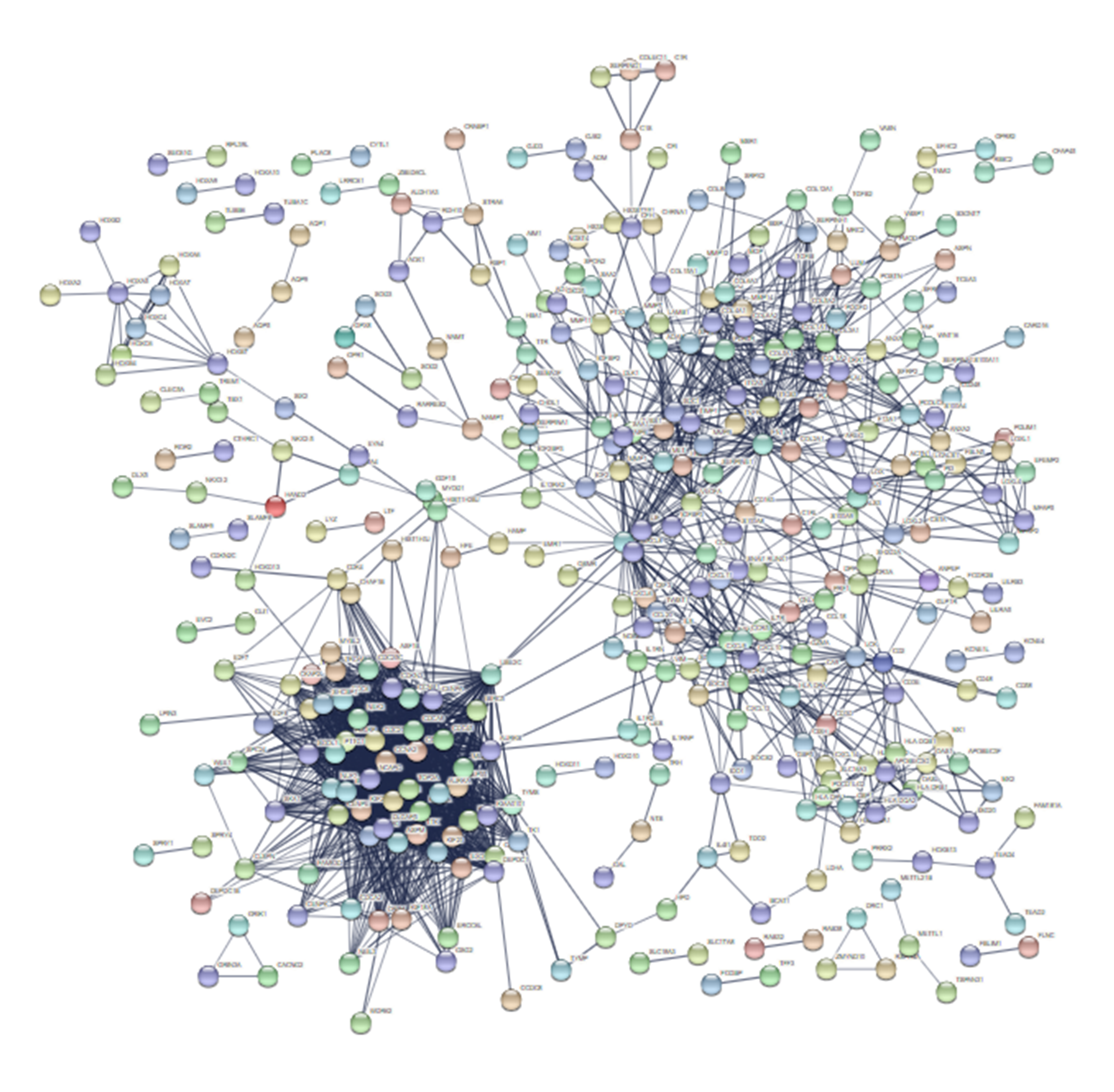

Supplement: Supplementary Figure 3 — The protein interaction diagram. [file Image_3.tif]
